# Non-EPI Vaccine Hesitancy among Chinese Adults: A Cross-Sectional Study

**DOI:** 10.3390/vaccines9070772

**Published:** 2021-07-10

**Authors:** Jianli Wang, Yan Zhang, Sigui Long, Xin Fu, Xiaoxuan Zhang, Shuangyu Zhao, Shixin Xiu, Xuwen Wang, Bing Lu, Hui Jin

**Affiliations:** 1 Department of Epidemiology and Health Statistics, School of Public Health, Southeast University, Nanjing 210009, China; 213171070@seu.edu.cn (J.W.); 213171093@seu.edu.cn (Y.Z.); 213180365@seu.edu.cn (S.L.); 213180456@seu.edu.cn (X.F.); 213181280@seu.edu.cn (X.Z.); 213181074@seu.edu.cn (S.Z.); 2 Key Laboratory of Environmental Medicine Engineering, School of Public Health, Southeast University, Ministry of Education, Nanjing 210009, China; 3 Wuxi Center for Disease Control and Prevention, Wuxi 214023, China; wxcdcsx@163.com (S.X.); wxmgwang@163.com (X.W.); Lub19661023@163.com (B.L.)

**Keywords:** Chinese adults, vaccine hesitancy, non-EPI vaccine, COVID-19 vaccine

## Abstract

Vaccination against coronavirus disease 2019 (COVID-19) is paramount to curtailing the pandemic. However, the impact of the Non-Expanded Program on Immunization (non-EPI) and COVID-19 vaccine hesitancy on vaccine uptake among Chinese adults remain unclear. This study was an online survey performed in Eastern, Central, and Western China between February 2021 and March 2021 using proportional sampling (*n* = 7381). Adults aged ≥ 18 years were included, especially younger people (aged < 65). Vaccine hesitancy was assessed using the 3C model and relative scales; logistic regression was used to explore the factors affecting vaccination uptake; structural equation modeling was used to evaluate the correlations between variables. Overall, 67.6% and 24.7% of adults reported vaccine hesitancy toward the non-EPI and COVID-19 vaccines, respectively. Participants (66.3%) reported taking the vaccine mainly based on recommendations from medical staff. Vaccine-hesitant participants (60.5%) reported a fear of side effects as the deciding factor in vaccine rejection. Vaccine hesitancy interacted negatively with confidence (β = −0.349, *p* < 0.001) and convenience (β = −0.232, *p* < 0.001), and positively with complacence (β = 0.838, *p* < 0.001). Nonmedical personnel, adults who had previously received the influenza vaccine, and older people had lower vaccine hesitancy than their counterparts. Most Chinese adults have non-EPI but not COVID-19 vaccine hesitancy. Vaccine safety remains a concern.

## 1. Introduction

Vaccine hesitancy involves denying or delaying vaccination when vaccination services are available while agreeing to receive other medical services [1]. Vaccine hesitancy affects vaccination coverage and may cause severe public health consequences. For example, global media misinformation about adverse reactions to the human papilloma virus (HPV) vaccine in 2013 resulted in the rates of this vaccine uptake dropping from 70% to 0.6% in Japan [2]. The belief that the measles, mumps, and rubella vaccines cause autism led to low vaccine coverage in Sweden [3]. Anti-vaccination campaigns in Malaysia have created high rates of vaccine hesitancy [4]. Similarly, Chinese people tend to delay or refuse vaccinations due to negative information about the risks of vaccines [5]. The World Health Organization identified vaccine hesitancy as among the top 10 global health threats in 2019 [6].

To address vaccine hesitancy, a “3Cs” model was proposed in 2011 [7], highlighting complacency, convenience, and confidence as the key drivers. Other relevant factors can fall into three groups [6]: social factors (history, society, culture, environment, health systems/institutions, economy, and politics); individual and group factors (personal views on vaccines); and direct factors associated with vaccines or vaccinations. Especially, age is one of the most important factors in view of decreased immunity and existing chronic diseases, and older adults tend to be more vulnerable to acquiring or developing more severe illnesses, along with life-threatening complications [8,9]. However, these illnesses can be prevented via vaccination programs.

Mass vaccinations with high coverage play an important role in disease outbreak control and prevention, helping curtail infection spread and build herd immunity. However, recent surveys have shown a high level of vaccine hesitancy worldwide [10]. A cross-sectional survey in the United States [11] and the UK [12] showed that the COVID-19 vaccine hesitation rates were about 50% and 40%, respectively. In fact, the rate of coronavirus disease 2019 (COVID-19) vaccine hesitancy in China is 20% [13,14]. Moreover, a longitudinal follow-up study between March and November to December 2020 showed that the willingness to receive a vaccination declined remarkably in China [15]. However, few studies have investigated the degree of COVID-19 vaccination hesitancy in the Chinese population since the outbreak of this disease; in addition, the factors driving vaccination hesitancy remain unclear. Despite the progress in COVID-19 vaccine efficacy and availability, vaccination hesitancy may reduce the effectiveness of the global immunization program.

This study aimed to examine the rate of vaccine hesitancy among adults in Eastern, Central, and Western China using online questionnaires; the factors associated with this hesitancy were also examined. This study focused on the Non-Expanded Program on Immunization (non-EPI) and COVID-19 vaccines. The present findings may inform immunization guidelines and policies, helping to reduce the rates of vaccination hesitancy and increase those of vaccination uptake.

## 2. Materials and Methods

This study was conducted in 31 provinces of Eastern, Central, and Western China (Supplementary Figure S1) between 9 February 2021 and 13 March 2021 using online questionnaires. We cooperated with some local disease control centers and hospitals. Moreover, our survey team members were distributed in almost all provinces, and each member collected a certain number of online questionnaires locally. Handy sampling was adopted; proportional sampling was performed per region. Sampling was conducted according to the population ratio of each province in the three regions and conducted in the form of an online anonymous survey through Questionnaire Star (the most widely used questionnaire platform in China). A total of 8256 questionnaires were collected in this study, and 7318 valid questionnaires were finally included in the study. Only Chinese adults aged ≥18 years were included. The following formula was used to calculate the sample size:

$$N_{min}=deff\times \frac{Z^{2}{}_{\left(1-\alpha /2\right)}\;\times \;p\;\times \;\left(1-p\right)}{d^{2}}$$

Type I error (α) was set to 0.05, precision (*d*) value was set to 0.04, and the desired effect size (*deff*) was set to 2 [14,15]. Vaccine hesitancy rate (95% confidence interval (CI)) (*p*) was conservatively set at 50%. The minimum sample size was estimated as 2081. For this cross-sectional survey, we set the sample size to 2200 people under the conditions of shedding and deletion. We estimated the required sample size to be 6600 (3 × 2200) participants, based on these calculations.

### 2.1. Measures

The questionnaire consisted of three parts: demographic characteristics, non-EPI vaccine hesitation, and COVID-19 vaccination willingness.

The demographic characteristics of interest were age, gender, ethnicity, place of residence, education, occupation, whether engaged with healthcare-related industries or not, annual family income, social contacts (including the number of people in residence and the number of daily contacts), and health behaviors, including self-reported health status and the history of influenza vaccination.

Based on the “3C” model and other models [16], a vaccine hesitancy survey scale was created comprising 15 items (Supplementary Table S1). In the end, 11 items were included (Supplementary Table S2) based on a reliability and validity analysis [17].

The COVID-19 vaccination willingness questionnaire included the willingness to get vaccinated, the causes of vaccination, and adverse reactions after vaccination. The willingness to vaccinate was measured using the following question: “Are you willing to take the COVID-19 vaccine?” The answers were “yes”, “not sure”, and “no.”

### 2.2. Statistical Analysis

Critical ratio, Spearman rank correlation coefficient, and internal consistency analysis were used to screen the survey items. Internal consistency reliability and split-half reliability were used to test the scale reliability; split-half reliability was tested using the odd–even method.

Content and construct validity were examined. Construct validity was evaluated by a confirmatory factor analysis, which examined whether the dimensions of a scale were consistent with the corresponding theoretical framework. An exploratory factor analysis was used with the adjusted scale. The maximum coefficient of variation method was used for orthogonal rotation (varimax) in the factor load matrix analysis (Supplementary Table S3). Moreover, a confirmatory factor analysis showed that the model with 11 items had a relatively better goodness-of-fit (Supplementary Table S4), with meaningful Cronbach’s alpha values for the confidence, complacency, and convenience dimensions and the overall scale (Supplementary Table S5); consequently, the 11-item scale was used [18].

SPSS (version 23.0, IBM, Armonk, NY, USA) and Microsoft Excel (version 2019, Microsoft Corp., Redmond, WA, USA) were used for the statistical analysis. For continuous variables, a single-factor analysis of variance or independent sample *t*-test were used for between-group comparisons of variables that met the normality of distribution and homogeneity of variance assumptions; the nonparametric rank sum test was used when these assumptions were not met. For categorical variable comparisons, the chi-square or Fisher’s exact tests were used. Odds ratios (ORs) and 95% confidence intervals (CIs) were used to explore the factors affecting COVID-19 vaccine uptake willingness using a logistic regression analysis. Structural equation modeling (SEM) was used to further study the factors affecting vaccine hesitancy (Supplementary Table S6). Statistical significance was set as two-sided *p*-values of <0.05. In this analysis, responses referring to “hesitation” and “uncertainty” were combined into a single “hesitation” item.

### 2.3. Factors Affecting Vaccine Hesitancy

Amos software (version 24.0, IBM, Armonk, NY, USA) was used to establish the 3C dimensions of vaccine hesitancy in a multifactorial SEM. We constructed a variable set based on the survey data and the “3C model.” “Confidence” included five items (Q1, Q2, Q3, Q4, and Q6); “complacency” included three items (Q8, Q9, and Q10); and “convenience” included Q12 and some items of Q13 and Q14. “Vaccine hesitation” included Q16 items. The assignment tables are presented in Table 1. The obtained model was modified through an adaptation test and Delphi Expert Consultation method [19]. Finally, we conducted a fractional analysis.

## 3. Results

A total of 7318 out of 8000 respondents were included in this study. Among them, 682 participants were excluded due to data quality concerns. Overall, 70.9% of the participants were younger people aged 18–35 years old, and older people merely accounted for very small proportion (Table 2); over 75% and 34.5% of the participants had a college or equivalent degree and an annual family income in the range of 50,000–100,000 RMB, respectively. Among the participants, 2616 (35.7%) were engaged with the enterprise/business/service industry, while 2386 (32.6%) were employees of the healthcare sector (including doctors, nurses, public health physicians, and pharmacy technicians, among others). Most (86.3%) respondents reported being in good health.

### 3.1. Non-EPI Vaccine Willingness among Adults

**Figure 1:**
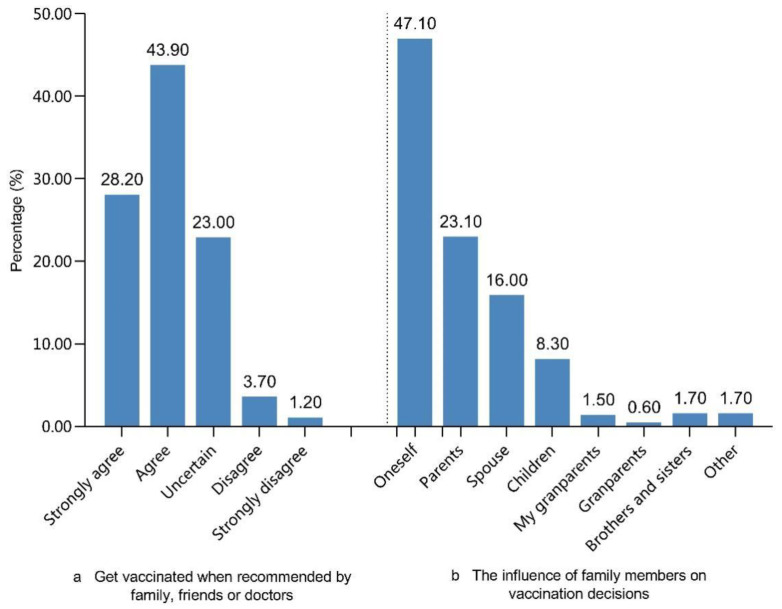
Impact of peer groups on vaccination attitudes. (**a**) Attitudes towards non-EPI vaccinations recommended by family, friends, or doctors. (**b**) Impact of family members on non-EPI vaccination decisions (N = 7318).

**Figure 2:**
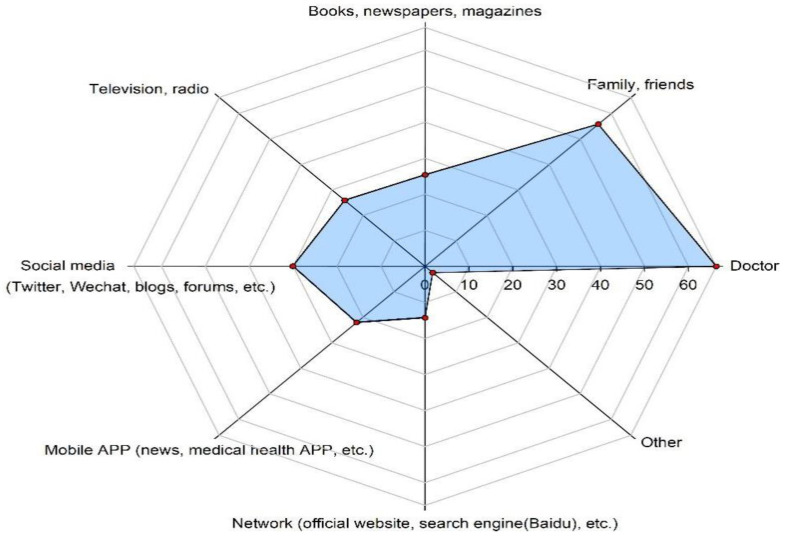
Primary sources of information on non-EPI vaccinations (N = 7318).

A total of 4945 (67.6%) participants reported hesitancy regarding the non-EPI vaccines (Table 2); in addition, 72.10% of the participants declared that “If family, friends, or doctors recommend vaccines to me, I’d be inclined to be vaccinated” (Figure 1a). Meanwhile, individual convictions (47.1%) and parental influence affected the decision to vaccinate (Figure 1b). The decision to receive a non-EPI vaccine was determined by sources of information, such as doctors (66.3%), family or friends (55.8%), and social media (30.1%; Figure 2). The findings from the Likert scale (Supplementary Table S1) indicated that over 60% of adults strongly agreed/trusted or agreed/trusted items Q1–Q7 and Q12–Q14; conversely, a quite low percentage (approximately 30%) showed agreement/trust in items Q9 and Q10 (Supplementary Figure S2).

### 3.2. Willingness to Take the COVID-19 Vaccine among Adults

**Figure 3:**
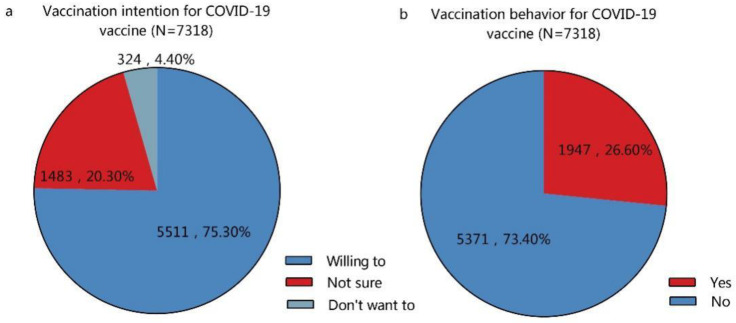
Participants declaring COVID-19 vaccination (**a**) intention and (**b**) uptake.

**Figure 4:**
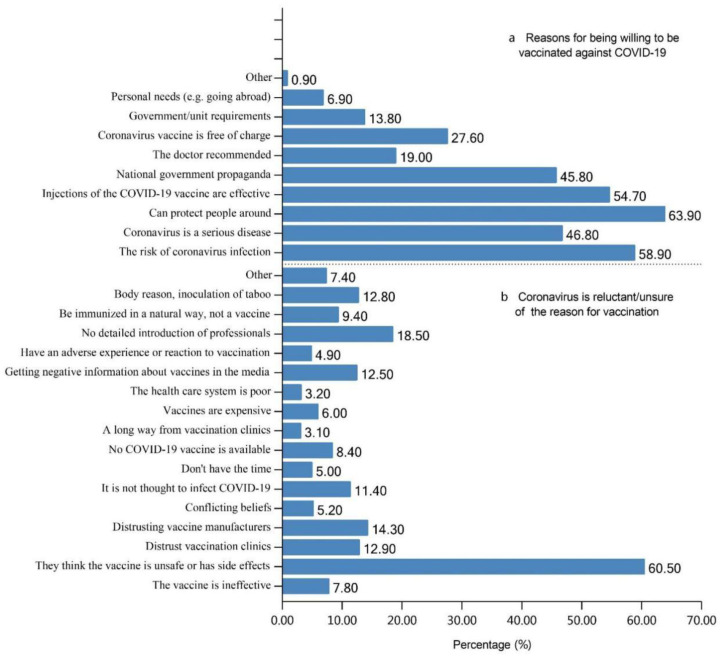
Reasons for COVID-19 vaccination (**a**) willingness and (**b**) refusal.

A total of 75.3% of the participants reported a willingness to accept the COVID-19 vaccine (Figure 3a), quoting “protecting others” (86.36%), “preventing infection” (58.9%), and believing that the “COVID-19 vaccine is effective” (54.70%) as the key motivators (Figure 4a). However, among 24.7% of the participants who declared vaccination unwillingness/refusal (Figure 3a), the most frequently (60.5%) cited reason was “concern about side effects and safety” (Figure 4b). A total of 26.60% of the participants had received the COVID-19 vaccine (Figure 3b); among them, 58% reported no adverse events, while 19.2% and 18.5% of the vaccinated participants reported symptoms of hypodynamia and localized pain, respectively (Supplementary Figure S3).

### 3.3. Factors Associated with Vaccine Hesitancy

The factors associated with vaccine hesitancy were younger age, higher education and annual income levels, lower number of daily contacts, better self-reported health status, working for sectors other than healthcare, and no history of influenza vaccination (*p* < 0.05) (Table 2). Furthermore, higher rates of COVID-19 vaccine hesitancy were observed among women and the residents of Eastern China than among their counterparts (*p* < 0.05).

The results of the multivariate analysis are presented in Supplementary Table S7. Participants with ≥21 daily contacts showed lower vaccine hesitancy compared with those with 1–10 daily contacts, and holders of college or equivalent degrees (OR: 0.752, 95% CI: 0.597–0.948, *p* = 0.016) were more willing to accept vaccinations compared with participants with junior high school and below education; soldiers (OR: 0.635, 95% CI: 0.56–0.719, *p* = 0.002) had lower vaccine hesitancy than those in government and institutions; compared with medical health practitioners, non-medical health practitioners (OR: 0.547, 95% CI: 0.371–0.807, *p* < 0.001) had lower rates of vaccine hesitancy. In contrast, higher rates of vaccine hesitancy were observed in participants with an average self-rated health status (OR: 1.538, 95% CI: 1.299–1.822, *p* < 0.001) and those that had never received the influenza vaccine (OR: 1.801, 95% CI: 1.591–2.038, *p* < 0.001), including production personnel in agriculture, forestry, animal husbandry, and fishery (OR: 1.255, 95% CI: 1.027–1.534, *p* = 0.026).

In addition, the participants residing in Central (OR: 0.681, 95% CI: 0.582–0.798, *p* < 0.001) and Western China (OR: 0.821, 95% CI: 0.705–0.955, *p* = 0.011) showed lower rates of COVID-19 vaccine hesitancy than those living in the eastern regions (Supplementary Table S7). Moreover, participants with ≥ 21 daily contacts (OR: 0.776, 95% CI: 0.642–0.938, *p* = 0.009) showed lower rates of COVID-19 vaccine hesitancy than their counterparts. The participants with a master’s or higher degree had a higher rate (OR: 1.372, 95% CI: 1.012–1.860, *p* = 0.041) of COVID-19 vaccine hesitancy than those with a junior high school or lower degree. Compared to those with a very good self-rated health status, all others with a good, common, or poor self-rated health status (except those with very poor one) were more likely to have COVID-19 vaccine hesitancy. The participants who had never received the influenza vaccine (OR: 2.9952, 95% CI: 2.536–3.539, *p* < 0.001) and nonhealthcare workers (OR: 1.426, 95% CI: 1.232–1.65, *p*< 0.001) showed higher rates of vaccine hesitancy than their counterparts. Moreover, higher rates of COVID-19 vaccine hesitancy were observed in adults with an annual household income of ≥160,000 RMB (OR: 1.465, 95% CI: 1.211–1.772, *p* < 0.001) than in those with annual household incomes of <50,000.

**Figure 5:**
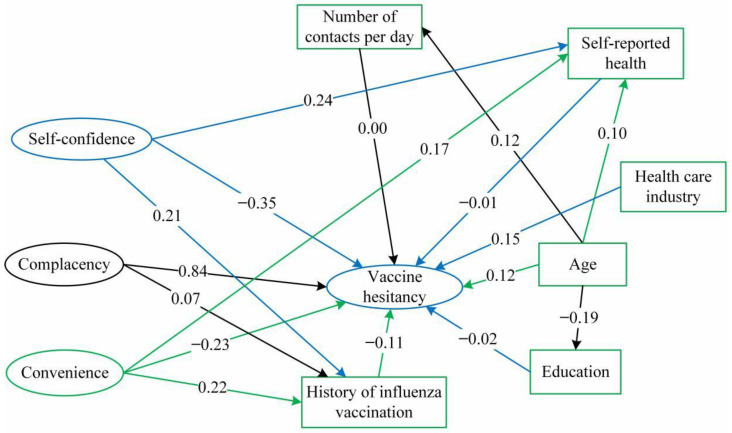
Structural equation model of vaccine hesitancy among adults.

The 3C dimensions, path diagram, and standardized path coefficient of the SEM of adult vaccine hesitancy are shown in Figure 5. Vaccination hesitancy interacted negatively with confidence (β = −0.349, *p* < 0.001) and convenience (β = −0.232, *p* < 0.001) and positively with complacence (β = 0.838, *p* < 0.001). Moreover, complacence correlated with convenience. Finally, older adults, non-medical health practitioners, and those with a history of influenza vaccination had lower rates of vaccine hesitancy than adults aged 18–25 years.

## 4. Discussion

The present findings indicate an overall positive attitude toward non-EPI vaccines; however, nearly two-thirds of the participants reported vaccine hesitancy. In the present study, occupation, place of residence, education level, health, and economic status were associated with vaccine hesitancy. Moreover, younger people showed a higher rate of vaccine hesitancy. Wang’s research showed similar results: that vaccine confidence rates increased with increasing age (*p* < 0.05) and older people (age ≥ 65) showed the lowest rate of COVID-19 vaccine hesitancy. Additionally, distrust in the vaccine, the delivery system, and the government were all associated with younger age, which contributed to vaccine hesitancy [20].

Multiple factors may determine vaccine hesitancy. A relatively low level of vaccine hesitancy in adults aged ≥ 25 years may be associated with individual experience and information that allows to make accurate judgments about the benefits and risks of vaccination. A higher income may also increase vaccine accessibility and convenience. Similarly, a lower vaccine hesitancy was observed among the members of the military, which might be relevant for the mobilization of groups. An atmosphere that facilitates vaccination may help improve the acceptance of vaccines by decreasing individual complacency [21]. A previous study showed that peer group support and mobilization from leaders may improve public trust in vaccines [22]. Participants with college or junior college degrees showed relatively lower rates of vaccine hesitancy than those who were less educated. In addition, people reportedly in good general health were likely to take vaccines for health protection. Adults with a number of daily contacts of ≥ 21 were less likely to refuse the non-EPI vaccines due to increased exposure to pathogens. Vaccinations boosts immunity and reduce the risk of disease.

Doctors and parents can influence attitudes toward vaccination uptake. The present study participants received their vaccination-related information mainly from doctors, family members, and friends, whose recommendations tended to increase the odds of becoming vaccinated. Physicians are among the most trusted sources of vaccine-related information [23]. However, in the present study, there was a relatively high rate of non-EPI vaccine hesitancy among medical staff, and this deserves closer examination; this finding may be associated with the improved sanitary conditions and lower risks of acquiring non-EPI vaccine-preventable diseases in this group. Vaccine hesitancy among healthcare staff has been previously reported [24,25] and should be addressed. In addition, parents play an important role in their children’s vaccination decisions. Chinese parents tend to have more confidence in the EPI than in non-EPI vaccines, which may be associated with their faith in governmental policy [13]. Thus, enhancing the education on non-EPI vaccines among adults, including parents, may increase the rates of vaccine uptake.

Most participants were willing to be vaccinated against COVID-19 to protect others and to prevent infection, believing the available vaccines to be effective; however, some participants refused to be vaccinated due to safety concerns, including the risk of adverse events; this finding is consistent with that of a previous study [26].

The risk of infection influenced the rate of COVID-19 vaccination. Health status and daily contact number affect COVID-19 vaccine hesitancy in a similar manner to non-EPI vaccines. Healthcare staff play a leading role in increasing the uptake of COVID-19 vaccines. Medical professionals involved in the treatment of COVID-19 patients were willing to be vaccinated against COVID-19, given the immunization’s role in the prevention of the disease [27,28]. Our study revealed a majority of healthcare workers (83%) were willing to accept the COVID-19 vaccine, while only 61% of Israeli nurses were willing to get vaccinated [29]. The low acceptance of the vaccine by nurses is not conducive to improving the vaccination confidence of the public. Healthcare staff should actively recommend vaccinations to the public [16] and provide accurate information on the safety and benefits of vaccines, which may help to improve public trust in vaccination programs. Medical personnel understand the benefits of vaccinations in disease prevention, resulting in relatively high uptake rates; the attitudes of healthcare workers may help set positive social norms and attitudes toward the COVID-19 vaccine, increasing the uptake. For instance, vaccinated health workers wearing badges that read “I have received vaccination to protect you from influenza” [30] may have contributed to the reduction in the incidence rate of pneumonia and influenza in the Netherlands [31]; this approach may also support COVID-19 vaccination uptake. Medical staff can help promote vaccinations; a comprehensive system of education, training, and incentives after inoculation may increase the uptake of vaccines [32].

Uptake of the COVID-19 vaccines is reduced by public fears of side effects. The COVID-19 vaccines do not trigger any adverse reactions among most vaccinated people; meanwhile, some people reported symptoms of asthenia or local pain, which raised public concern about vaccine safety. Government health departments should construct a transparent, robust, and reasonable immunization procedure for the COVID-19 vaccines and communicate the vaccine development process to the public [33] through context-specific campaigns, addressing the key concerns. Behavior change theories, such as the Health Belief Model and social marketing [34,35], may also help improve vaccination rates. High rates of COVID-19 vaccine hesitancy were observed among women; this finding was consistent with those of studies in the American and British populations [36,37]. The impact of gender-specific interventions requires further investigation.

Education and socioeconomic status, unlike the social determinants of health, had a dual impact on vaccine hesitancy in participants of different backgrounds. Higher levels of education (master’s degree or above) were associated with a lower rate of vaccine acceptance [38,39]. Higher levels of COVID-19 vaccine hesitancy were observed in participants with graduate degrees; this finding may be associated with excessive concerns over vaccine safety in this group. In addition, compared to the residents of Western and Central China, the residents of the more economically developed eastern areas, where household incomes are relatively high, reported some reluctance toward COVID-19 vaccine uptake. The intention to receive a COVID-19 vaccine was weak, regardless of the ability to access the relevant services and cover the associated costs.

This study has some limitations. First, the use of online questionnaires may have resulted in a sampling bias. In the present sample, 70.9% of adults were aged 18–35 years; older adults were under-represented. As the age of the individuals included in the study is a significant factor of vaccine hesitancy and older people are a population with specific characteristics, a further analysis of vaccine hesitancy in older people will be seriously considered and conducted in the future. Simultaneously, this sample lacked representativeness from some provinces; consequently, the present findings should be interpreted with caution. Second, the present study was based on self-reported information; thus, the results may be subject to self-reporting bias. Although the present findings suggest variations in the rates of COVID-19 vaccine acceptance among the residents of different parts of China, further studies are required to validate these findings, which may have resulted from the study design. Third, vaccine hesitancy and willingness are influenced by many complex factors, including timing and policy; a careful examination of the impact of survey methodology on the assessment of the attitudes toward vaccines is required. Despite these limitations, the present findings advance the understanding of vaccine hesitancy and provide guidance for interventions. The reasons behind a high level of vaccine hesitancy in non-EPI vaccines and a low level in COVID-19 vaccines among medical staff will be an important direction and hotspot for addressing vaccine hesitancy in the future.

## 5. Conclusions

Most Chinese adults present with non-EPI vaccine hesitancy, while some of them also present with COVID-19 vaccine hesitancy. In the present study, medical staff showed a relatively high rate of non-EPI vaccine hesitancy while a low rate of COVID-19 vaccine hesitancy, as the results showed that doctors are the main information source regarding vaccines and play a leading role in increasing the uptake of COVID-19 vaccines; greater awareness and targeted education of medical staff on disease risk and vaccine benefits may be required to increase vaccination coverage. Moreover, the present findings suggest that people who were confident and willing to receive the vaccination might not necessarily choose to be vaccinated; vaccine hesitancy was the key factor preventing people from receiving vaccinations. Interventions are required to address vaccine hesitancy and increase vaccine uptake among Chinese adults. Mass vaccination is crucial to curtailing the COVID-19 pandemic.

## Figures and Tables

**Table 1:** Variables assignment for the structural equation model of a vaccine.

Variable Meaning	Assignment
Q1. Generally, I think the vaccine is safe Q2. Generally, I think the vaccine is effective Q3. I think vaccines are very important for my health Q4. Generally, I think the whole chain (whole process, from production to vaccination) management of vaccine is safe and effective Q6. Please evaluate your trust in the vaccination information provided by the government Q8. In my opinion, immunity from natural diseases is better than vaccination Q9. Because of the low risk of disease, there is no need for vaccination Q10. Even if I have a disease, I can resist it, so I don’t need a vaccine Q12. Generally, it’s very convenient and time-consuming for me to get vaccinated Q13. Generally, I was able to get the vaccine I wanted Q14. Generally, I can afford the vaccine	1 = very agree; 2 = agree; 3 = uncertain; 4 = disagree; 5 = very disagree
Vaccination hesitancy	1 = very hesitant; 2 = hesitant; 3 = uncertain; 4 = not hesitant; 5 = very not hesitant

**Table 2:** Non-EPI vaccine hesitancy and COVID-19 vaccine hesitancy among participants with different demographics.

Demographic Characteristics	Number	Proportion (%)	Non-EPI Vaccine Hesitancy			COVID-19 Vaccine Hesitancy		
			Yes Frequency (%)	No Frequency (%)	*p* Value	Yes Frequency (%)	No Frequency (%)	*p* Value
Age, years								
18~25	2409	32.90	1771 (73.5)	638 (26.5)	<0.001	600 (24.9)	1809 (75.1)	<0.001
26~35	2784	38.00	1814 (65.2)	970 (34.8)		615 (22.1)	2169 (77.9)	
36~45	1264	17.30	811 (64.2)	453 (35.8)		320 (25.3)	944 (74.7)	
≥46	861	11.80	549 (63.8)	312 (36.2)		272 (31.6)	589 (68.4)	
Gender								
Male	3565	48.70	2382 (66.8)	1183 (33.2)	0.178	741 (20.8)	2824 (79.2)	<0.001
Female	3753	51.30	2563 (68.3)	1190 (31.7)		1066 (28.4)	2687 (71.6)	
Nationality								
Han	6979	95.40	4714 (67.5)	2265 (32.5)	0.819	1719 (24.6)	5260 (75.4)	0.580
Non-Han	339	4.60	231 (68.1)	108 (31.9)		88 (26.0)	251 (74.0)	
Place of residence								
East	4292	58.60	2938 (68.5)	1354 (31.5)	0.160	1160 (27.0)	3132 (73.0)	<0.001
Central	1559	21.30	1034 (66.3)	525 (33.7)		277 (17.8)	1282 (82.2)	
West	1467	20.00	973 (66.3)	494 (33.7)		370 (25.2)	1097 (74.8)	
Education level								
≤Junior high school	436	6.00	310 (71.1)	126 (28.9)	0.041	103 (23.6)	333 (76.4)	<0.001
High school or equivalent	1193	16.30	786 (65.9)	407 (34.1)		246 (20.6)	947 (79.4)	
College or equivalent	4692	64.10	3147 (67.1)	1545 (32.9)		1148 (24.5)	3544 (75.5)	
≥Master’s degree	997	13.60	702 (70.4)	295 (29.6)		310 (31.1)	687 (68.9)	
Occupation								
Government agencies and institutions								
	1681	23.00	1088 (64.7)	593 (35.3)	<0.001	415 (24.7)	1266 (75.3)	<0.001
Enterprise/business/service industry								
	2616	35.70	1724 (65.9)	892 (34.1)		561 (21.4)	2055 (78.6)	
Agricultural/forestry/animal husbandry/fishery/water conservancy production personnel								
	765	10.50	526 (68.8)	239 (31.2)		125 (16.3)	640 (83.7)	
Soldier	123	1.70	60 (48.8)	63 (51.2)		13 (10.6)	110 (89.4)	
Full-time student	1326	18.10	981 (74.0)	345 (26.0)		402 (30.3)	924 (69.7)	
Other	807	11.00	566 (70.1)	241 (29.9)		291 (36.1)	516 (63.9)	
Healthcare occupation								
Yes	2386	32.60	1666 (69.8)	720 (30.2)	0.004	402 (16.8)	1984 (83.2)	<0.001
No	4932	67.40	3279 (66.5)	1653 (33.5)		1405 (28.5)	3527 (71.5)	
Annual family income (RMB 10,000)								
<5	1481	20.20	1045 (70.6)	436 (29.4)	0.005	333 (22.5)	1148 (77.5)	<0.001
5–10	2524	34.50	1656 (65.6)	868 (34.4)		587 (23.3)	1937 (76.7)	
11–15	1738	23.70	1157 (66.6)	581 (33.4)		359 (20.7)	1379 (79.3)	
≥16	1575	21.50	1087 (69.0)	488 (31.0)		528 (33.5)	1047 (66.5)	
Number of people in residence								
1	477	6.50	329 (69.0)	148 (31.0)	0.364	121 (25.4)	356 (74.6)	0.752
2–5	6466	88.40	4374 (67.6)	2092 (32.4)		1599 (24.7)	4867 (75.3)	
≥6	375	5.10	242 (64.5)	133 (35.5)		87 (23.2)	288 (76.8)	
Number of contacts per day								
1–10	4684	64.00	3176 (67.8)	1508 (32.2)	0.002	1224 (26.1)	3460 (73.9)	0.001
11–20	1824	24.90	1264 (69.3)	560 (30.7)		400 (21.9)	1424 (78.1)	
≥21	810	11.10	505 (62.3)	305 (37.7)		183 (22.6)	627 (77.4)	
Self-reported health								
Very good	3508	47.90	2275 (64.9)	1233 (35.1)	<0.001	614 (17.5)	2894 (82.5)	<0.001
Good	2813	38.40	1929 (68.6)	884 (31.4)		848 (30.1)	1965 (69.9)	
Common	921	12.60	688 (74.7)	233 (25.3)		320 (34.7)	601 (65.3)	
Bad	56	0.80	43 (76.8)	13 (23.2)		20 (35.7)	36 (64.3)	
Very bad	20	0.30	10 (50.0)	10 (50.0)		5 (25.0)	15 (75.0)	
Have you ever been obtained the influenza vaccination?								
Yes	2293	31.30	1398 (61.0)	895 (39.0)	<0.001	223 (9.7)	2070 (90.3)	<0.001
No	5025	68.70	3547 (70.6)	1478 (29.4)		1584 (31.5)	3441 (68.5)	
**Total**	7318	100.00	4945 (67.6)	2373 (32.4)		1807 (24.7)	5511 (75.3)	

## Data Availability

The data presented in this study are available on request from the corresponding author.

## Supplementary Materials

The following are available online at https://www.mdpi.com/article/10.3390/vaccines9070772/s1: Supplementary Table S1. Fifteen-item Likert scale of vaccine hesitancy. Supplementary Table S2. Item selection in the development of the vaccine hesitancy scale. Supplementary Table S3. Factor-loading matrices in the exploratory factor analysis. Supplementary Table S4. The reliability of the vaccine hesitancy scale. Supplementary Table S5. Model fit indexes in the confirmatory factor analysis. Supplementary Table S6. Fitness test in the multivariate structural equation model of vaccine hesitancy among adults. Supplementary Table S7. Multivariate analysis of the factors affecting non-EPI and COVID-19 vaccine hesitancy. Figure S1. The thirty-one provinces surveyed in China. Supplementary Figure S2. Distribution of the participants’ responses to 15 items on the vaccine hesitancy scale. Supplementary Figure S3. Rates of adverse reactions to the COVID-19 vaccine.
