# Supplementary material for: Non-EPI Vaccine Hesitancy among Chinese Adults: A Cross-Sectional Study
Source: Vaccines (Basel). 2021 Jul 10;9(7):772. doi: 10.3390/vaccines9070772 (PMC8310190; doi:10.3390/vaccines9070772)
Supplement: Supplementary file 1 [file vaccines-09-00772-s001.zip › Supplementary Table S1.pdf]

Supplemental Table S1. Fifteen-item Likert scale of vaccine hesitancy

No.	Content
1	Generally, I think the vaccine is safe
2	Generally, I think the vaccine is effective
3	I think vaccines are very important for my health
4	Generally, I think the whole chain (whole process, from production to vaccination) management of vaccine is safe and effective
5	Please evaluate the degree of vaccine information and service trust you have provided to:
5.1	Doctor or nurse
5.2	Centers for disease control, hospitals, vaccination clinics or other
5.3	Vaccine manufacturers and companies
6	Please evaluate your trust in the vaccination information provided by the government
7	I think if I don't get the vaccine, I may get the disease
8	In my opinion, immunity from natural diseases is better than vaccination
9	Because of the low risk of disease, there is no need for vaccination
10	Even if I have a disease, I can resist it, so I don't need a vaccine
11	Generally,the poor service quality of vaccination clinic will make me not want to vaccinate again
12	Generally,it's very convenient and time-consuming for me to get vaccinated
13	Generally,I was able to get the vaccine I wanted
14	Generally,I can afford the vaccine
15	Generally,it would be good for me not to get vaccinated
	(e.g. don't spend money,don't worry about side effects of vaccine, etc.)
