# Supplementary material for: Non-EPI Vaccine Hesitancy among Chinese Adults: A Cross-Sectional Study
Source: Vaccines (Basel). 2021 Jul 10;9(7):772. doi: 10.3390/vaccines9070772 (PMC8310190; doi:10.3390/vaccines9070772)
Supplement: Supplementary file 1 [file vaccines-09-00772-s001.zip › Supplementary Table S2.pdf]

Supplemental Table S2. Item selection in the development of the vaccine hesitancy scale

Dimension	Item	Critical ratio method p value	Correlation coefficient with total score	Cronbach's α coefficient after deleting the item
Confidence	Q1.Generally, I think the vaccine is safe	<0.001	0.853**	0.670
	Q2.Generally, I think the vaccine is effective		0.856**	0.671
	Q3.I think vaccines are very important for my health		0.854**	0.669
	Q4.Generally, I think the whole chain (whole process, from production to vaccination) management of vaccine is safe and effective		0.849**	0.672
	Q6.Please evaluate your trust in the vaccination information provided by the government		0.789**	0.674
Complacent	Q7.I think if I don't get the vaccine, I may get the disease		-0.017	0.703
	Q8.In my opinion, immunity from natural diseases is better than vaccination		0.760**	0.699
	Q9.Because of the low risk of disease, there is no need for vaccination		0.872**	0.671
	Q10.Even if I have a disease, I can resist it, so I don't need a vaccine		0.866**	0.684
Convenient	Q11.Generally, the poor service quality of vaccination clinic will make me not want to vaccinate again		0.536**	0.686
	Q12.Generally, it's very convenient and time-consuming for me to get vaccinated		0.516**	0.687
	Q13.Generally, I was able to get the vaccine I wanted		0.502**	0.687
	Q14.Generally, I can afford the vaccine		0.508**	0.684
	Q15.Generally,it would be good for me not to get vaccinated (e.g. don't spend money,don't worry about side effects of vaccine, etc.)		0.431**	0.692

** represents P<0.001
