# Supplementary material for: Non-EPI Vaccine Hesitancy among Chinese Adults: A Cross-Sectional Study
Source: Vaccines (Basel). 2021 Jul 10;9(7):772. doi: 10.3390/vaccines9070772 (PMC8310190; doi:10.3390/vaccines9070772)
Supplement: Supplementary file 1 [file vaccines-09-00772-s001.zip › Supplementary Table S3.pdf]

Supplemental Table S3. Factor-loading matrices in exploratory factor analysis

Entry	Factor 1	Factor 2	Factor 3
Q1	0.851	-0.067	0.192
Q2	0.864	-0.072	0.163
Q3	0.848	-0.061	0.190
Q4	0.814	-0.098	0.247
Q6	0.735	-0.036	0.255
Q8	-0.148	0.747	-0.097
Q9	0.047	0.866	-0.115
Q10	-0.044	0.882	-0.148
Q11	-0.082	0.772	0.026
Q12	0.305	-0.155	0.763
Q13	0.301	-0.159	0.797
Q14	0.224	-0.063	0.799
Q15	-0.071	0.758	-0.115
