# Supplementary material for: Non-EPI Vaccine Hesitancy among Chinese Adults: A Cross-Sectional Study
Source: Vaccines (Basel). 2021 Jul 10;9(7):772. doi: 10.3390/vaccines9070772 (PMC8310190; doi:10.3390/vaccines9070772)
Supplement: Supplementary file 1 [file vaccines-09-00772-s001.zip › Supplementary Table S4.pdf]

Supplemental Table S4. The reliability of the vaccine hesitancy scale

Dimension	The number of items	Cronbach's α
Full scale	11	0.688
Confidence	5	0.906
Complacement	3	0.853
Convenience	3	0.798
