# Supplementary material for: Non-EPI Vaccine Hesitancy among Chinese Adults: A Cross-Sectional Study
Source: Vaccines (Basel). 2021 Jul 10;9(7):772. doi: 10.3390/vaccines9070772 (PMC8310190; doi:10.3390/vaccines9070772)
Supplement: Supplementary file 1 [file vaccines-09-00772-s001.zip › Supplementary Table S5.pdf]

Supplementary Table S5. Model fit indexes in confirmatory factor analysis

Model	$\chi2$	df	$\chi2$/df	RMSEA	GFI	AGFI
13 items	7565.323	62	122.021	0.129	0.851	0.782
Exxcept Q11	4052.476	51	79.460	0.104	0.925	0.885
Except Q11\15	861.624	41	21.015	0.052	0.979	0.966
