# Supplementary material for: Non-EPI Vaccine Hesitancy among Chinese Adults: A Cross-Sectional Study
Source: Vaccines (Basel). 2021 Jul 10;9(7):772. doi: 10.3390/vaccines9070772 (PMC8310190; doi:10.3390/vaccines9070772)
Supplement: Supplementary file 1 [file vaccines-09-00772-s001.zip › Supplementary Table S6.pdf]

Supplementary Table S6. Fitness test in the multivariate structural equation model of vaccine hesitancy among adults

Index	RMSEA	GFI	AGFI	NFI	CFI
Model matching degree	0.054	0.945	0.924	0.760	0.768
Recommended value	<0.06	>0.90	>0.90	>0.90	>0.90
