# Supplementary material for: Non-EPI Vaccine Hesitancy among Chinese Adults: A Cross-Sectional Study
Source: Vaccines (Basel). 2021 Jul 10;9(7):772. doi: 10.3390/vaccines9070772 (PMC8310190; doi:10.3390/vaccines9070772)
Supplement: Supplementary file 1 [file vaccines-09-00772-s001.zip › Supplementary Table S7.pdf]

Supplemental Table S7. Multivariate analysis of factors affecting non-EPI and COVID-19 vaccine hesitancy

Demographic characteristics		Non-EPI vaccines		COVID-19 vaccines	
		OR, 95%*CI*	*P*-value	*OR*, 95%*CI*	*P*-value
**Age**	18-25（Reference）				
	26-35	0.683(0.590,0.790)	0.000	-	-
	≥46	0.536(0.439,0.654)	0.000	-	-
**Educational level**	Junior high school or below (Reference)				
	High school graduat or equivalent	0.802(0.627,1.027)	0.080	1.103(0.831,1.464)	0.497
	College or equivalent	0.752(0.597,0.948)	0.016	1.075(0.825,1.401)	0.591
	Master's Diploma or above	0.894(0.685,1.166)	0.408	1.372(1.012,1.860)	0.041
**Occupation**	Government agencies and institutions（Reference）				
	Enterprise / business / service industry	1.153(1.000,1.328)	0.050	0.888(0.753,1.047)	0.158
	Agricultural, forestry, animal husbandry, fishery and water conservancy production personnel	1.255(1.027,1.534)	0.026	0.946(0.737,1.215)	0.665
	Soldier	0.547(0.371,0.807)	0.002	0.957(0.514,1.783)	0.890
	Full-time student	1.115(0.909,1.367)	0.297	1.124(0.935,1.35)	0.214
	Other	1.248(1.026,1.517)	0.026	1.574(1.283,1.932)	0.000
**Number of contacts per day**	1-10（Reference）				
	11-20	1.158(1.024,1.310)	0.019	1.046(0.909,1.205)	0.529
	≥21	0.817(0.695,0.960)	0.014	0.776(0.642,0.938)	0.009
**Self-reported health**	Very good（Reference）				
	Good	1.112(0.995,1.243)	0.062	1.484(1.308,1.684)	0.000
	Common	1.538(1.299,1.822)	0.000	2.014(1.698,2.389)	0.000
	Bad	1.586(0.843,2.983)	0.152	2.496(1.390,4.484)	0.002
	Very bad	0.504(0.205,1.236)	0.134	1.505(0.510,4.443)	0.459
**Having influenza vaccination**	Yes				
	No	1.801(1.591,2.038)	0.000	2.995(2.536,3.539)	0.000
**Healthcare occupation**	Yes				
	No	0.635(0.56,0.719)	0	1.426(1.232,1.651)	0.000
**Place of abode**	East（Reference）				
	Central	-	-	0.681(0.582,0.798)	0.000
	West	-	-	0.821(0.705,0.955)	0.011
**Annual household income (RMB 10,000)**	<5（Reference）				

	5-10	-	-	1.029(0.870,1.216)	0.740
	11-15	-	-	0.922(0.762,1.114)	0.399
	≥16	-	-	1.465(1.211,1.772)	0.000
