# Supplementary figures and images for: Non-EPI Vaccine Hesitancy among Chinese Adults: A Cross-Sectional Study
Source: Vaccines (Basel). 2021 Jul 10;9(7):772. doi: 10.3390/vaccines9070772 (PMC8310190; doi:10.3390/vaccines9070772)

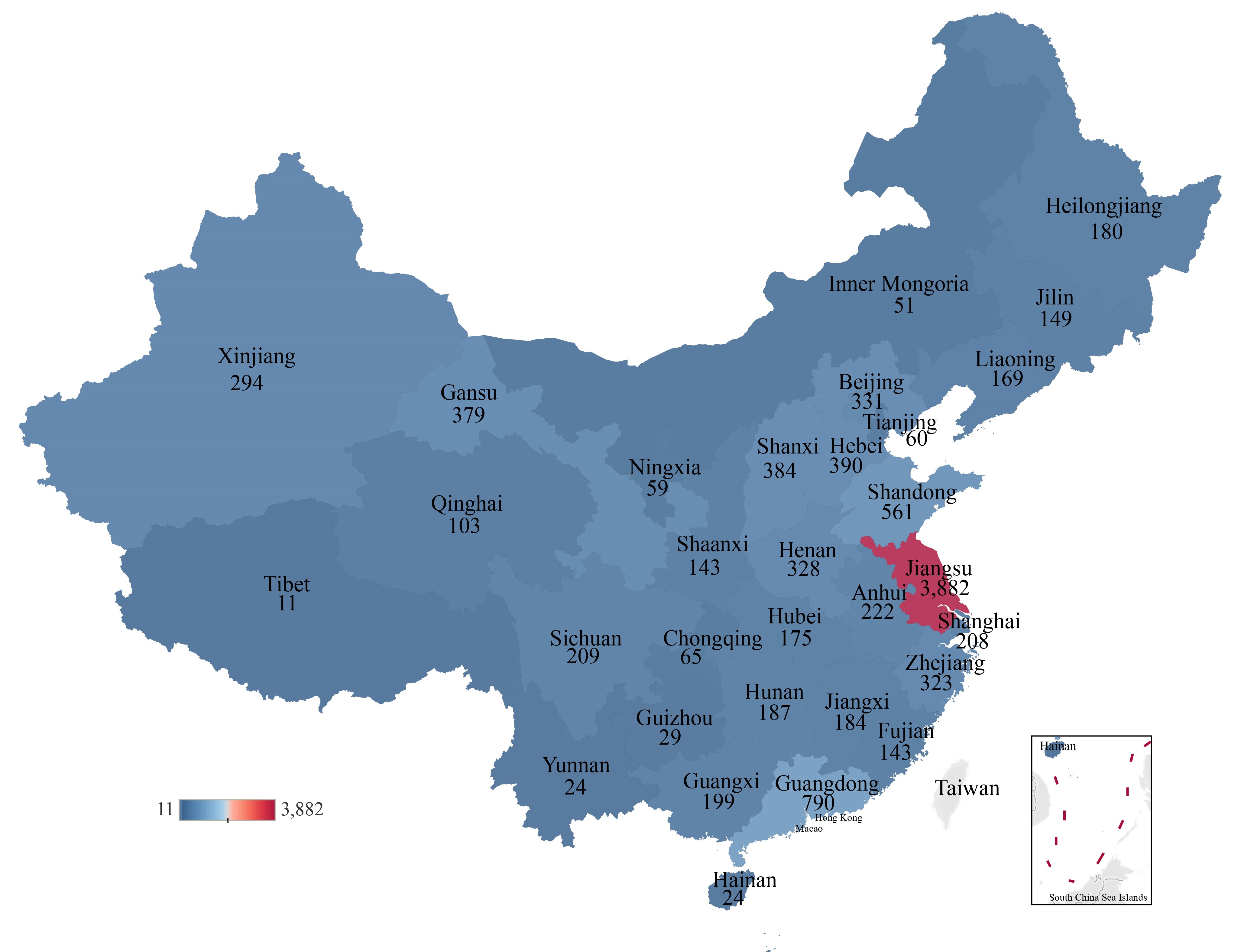

Supplement: Supplementary file 1 [file vaccines-09-00772-s001.zip › Supplementary Figure S1.jpg]

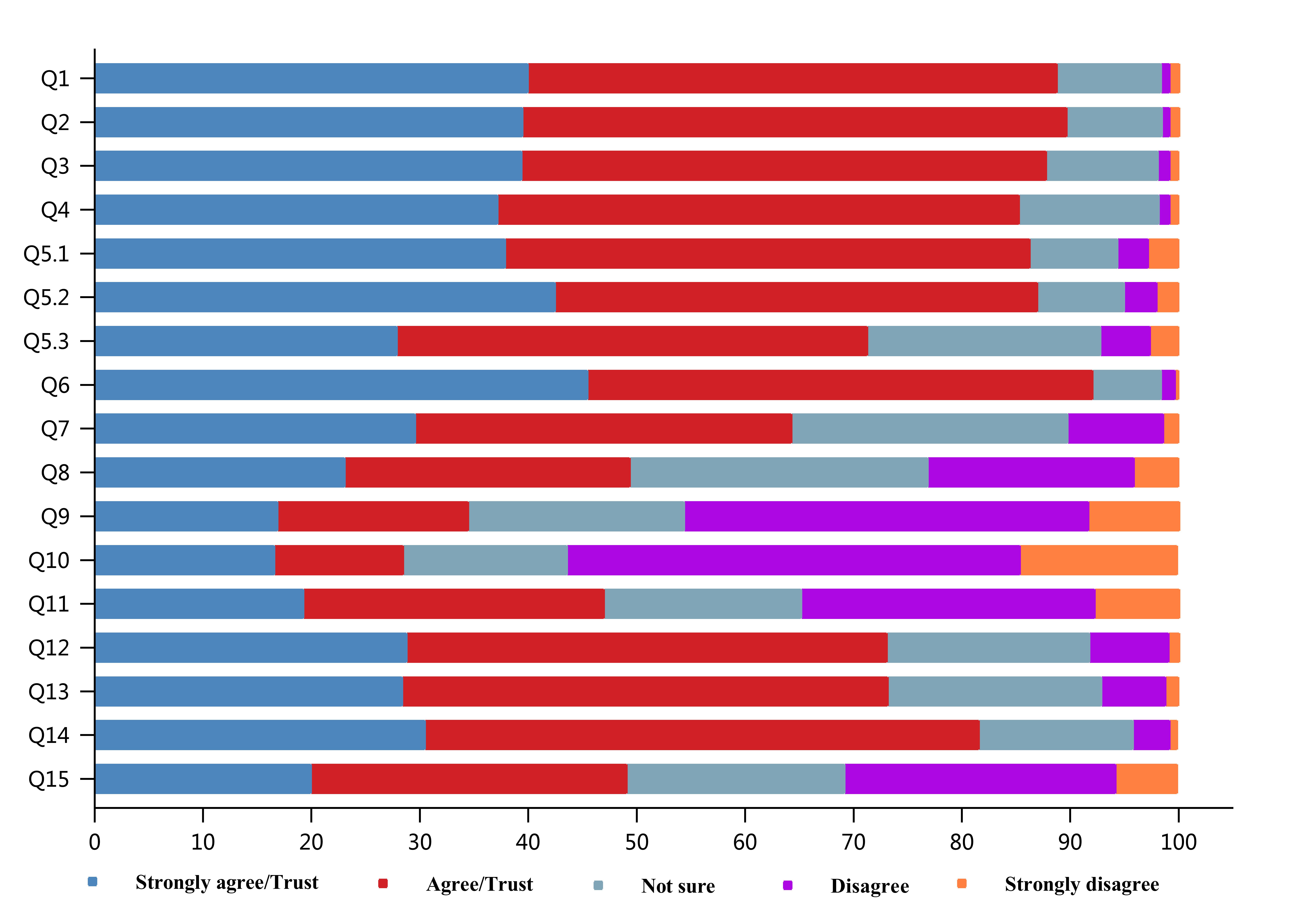

Supplement: Supplementary file 1 [file vaccines-09-00772-s001.zip › Supplementary Figure S2.jpg]

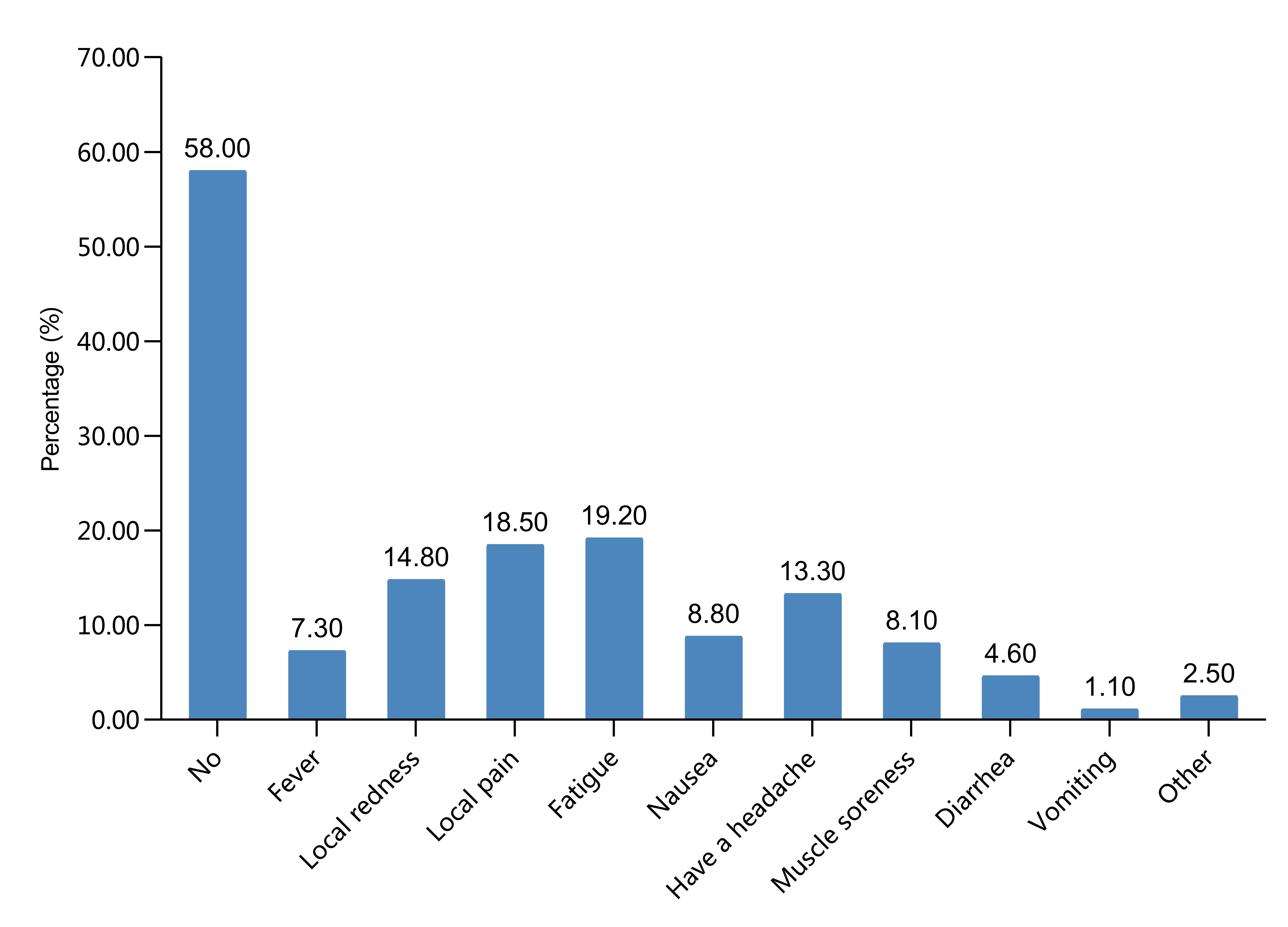

Supplement: Supplementary file 1 [file vaccines-09-00772-s001.zip › Supplementary Figure S3.jpg]
